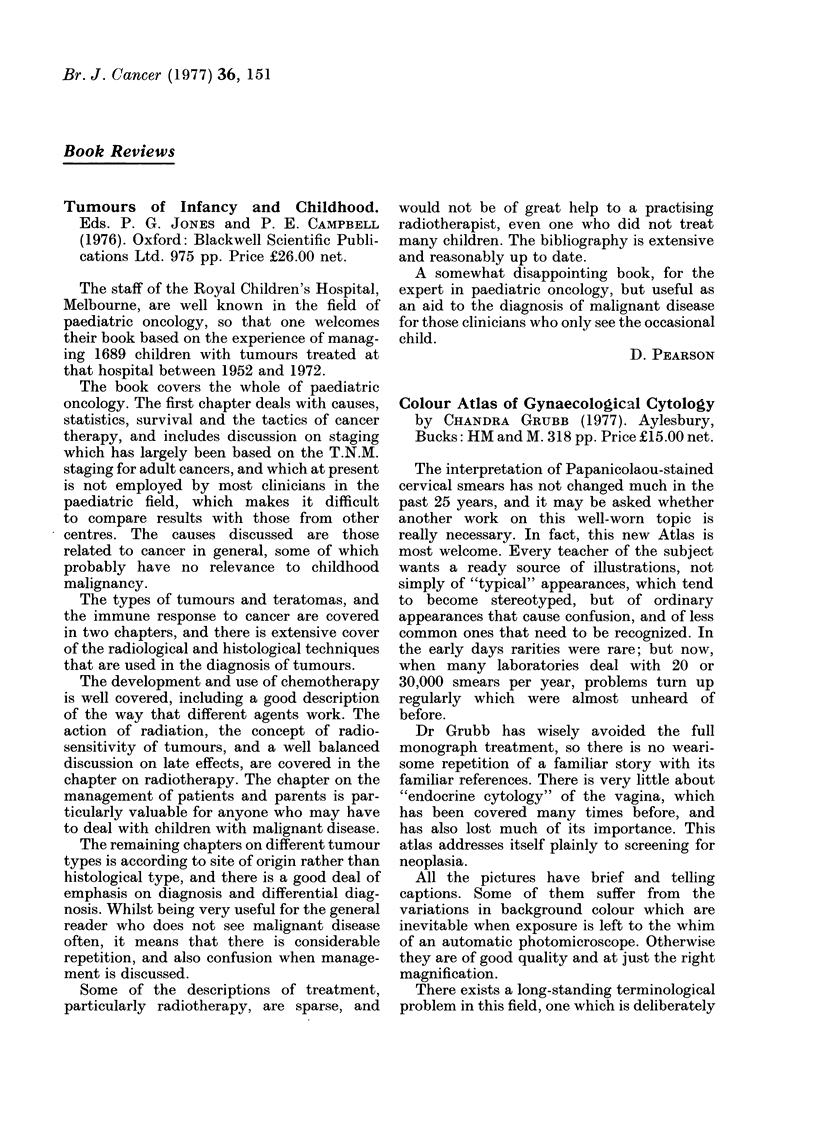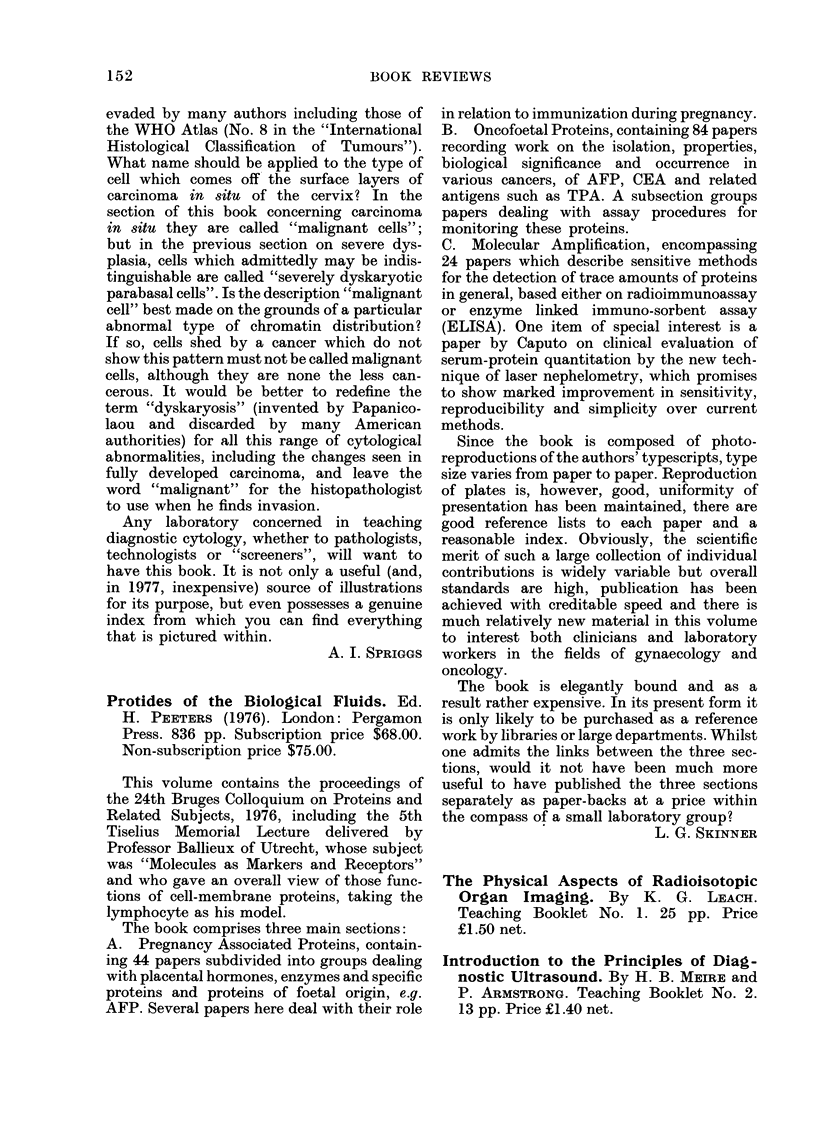# Colour Atlas of Gynaecological Cytology

**Published:** 1977-07

**Authors:** A. I. Spriggs


**Colour Atlas of Gynaecological Cytology** by Chandra Grubb (1977). Aylesbury, Bucks: HM and M. 318 pp. Price £15.00 net.

The interpretation of Papanicolaou-stained cervical smears has not changed much in the past 25 years, and it may be asked whether another work on this well-worn topic is really necessary. In fact, this new Atlas is most welcome. Every teacher of the subject wants a ready source of illustrations, not simply of "typical" appearances, which tend to become stereotyped, but of ordinary appearances that cause confusion, and of less common ones that need to be recognized. In the early days rarities were rare; but now, when many laboratories deal with 20 or 30,000 smears per year, problems turn up regularly which were almost unheard of before.

Dr Grubb has wisely avoided the full monograph treatment, so there is no wearisome repetition of a familiar story with its familiar references. There is very little about "endocrine cytology" of the vagina, which has been covered many times before, and has also lost much of its importance. This atlas addresses itself plainly to screening for neoplasia.

All the pictures have brief and telling captions. Some of them suffer from the variations in background colour which are inevitable when exposure is left to the whim of an automatic photomicroscope. Otherwise they are of good quality and at just the right magnification.

There exists a long-standing terminological problem in this field, one which is deliberately evaded by many authors including those of the WHO Atlas (No. 8 in the "International Histological Classification of Tumours"). What name should be applied to the type of cell which comes off the surface layers of carcinoma *in situ* of the cervix? In the section of this book concerning carcinoma *in situ* they are called "malignant cells"; but in the previous section on severe dysplasia, cells which admittedly may be indistinguishable are called "severely dyskaryotic parabasal cells". Is the description "malignant cell" best made on the grounds of a particular abnormal type of chromatin distribution? If so, cells shed by a cancer which do not show this pattern must not be called malignant cells, although they are none the less cancerous. It would be better to redefine the term "dyskaryosis" (invented by Papanicolaou and discarded by many American authorities) for all this range of cytological abnormalities, including the changes seen in fully developed carcinoma, and leave the word "malignant" for the histopathologist to use when he finds invasion.

Any laboratory concerned in teaching diagnostic cytology, whether to pathologists, technologists or "screeners", will want to have this book. It is not only a useful (and, in 1977, inexpensive) source of illustrations for its purpose, but even possesses a genuine index from which you can find everything that is pictured within.

A. I. Spriggs